# Therapeutic Nihilism: Is It Ignorance or Egotism?

**Published:** 1908-12

**Authors:** H. L. Henderson

**Affiliations:** Astoria, Oregon


### TEXAS MEDICAL JOURNAL.

Established July, 1885.

F. E. DANIEL, M. D., - - - - Editor, Publisher and Proprietor.

PUBLISHED MONTHLY.—SUBSCRIPTION $1.00 A YEAR.

VOL. XXIV. AUSTIN, DECEMBER, 1908. No. 6.

The publisher is not responsible for the views of contributors.

### Original Articles.

For Texas Medical Journal.

### Therapeutic Nihilism: Is It Ignorance or Egotism?

BY H. L. HENDERSON, M. D., ASTORIA, OREGON.

In the October issue of the TEXAS MEDICAL JOURNAL, the "Red Back," there appeared an article from one of the foremost physicians of the United States under the title, "The Auto-Protective Resources of the Body—A New Foundation for Scientific Therapeutics," from the pen of Charles E. De M. Sajous, M. D., of Philadelphia. At this juncture, I would strongly urge every reader of these lines to at once reread that article, carefully and critically, before continuing the perusal of this one? The article mentioned is one marked by an extreme degree of scholarship, together with a deep insight into and sympathy with the ills to which humanity is heir. I do not desire, at this time, to in any way detract from the latter portion of that able essay, nor, in fact, to detract from any part of it, but I do wish to call the attention of all thinking physicians to many of the quotations and inferences that would naturally follow after reading the first portion of the essay. As to the "Sajous Theory," the primary object for which the article was written, on that I have no comments to offer nor criticisms to promulgate. The first portion of the article brings out, prominently, that terrible disgrace of the medical profession, "Therapeutic Nihilism." It is surely time that the thinking portion of our profession called a halt, and after carefully examining the situation affix the proper label to that form of imbecility that is just at this time masquerading under the euphonious and high-sounding and misapplied term, "Therapeutic Nihilism." Would it not be thought strange if we should hear the ardent and devout minister of the gospel stand in his pulpit and loudly proclaim that he was a confirmed atheist or infidel? Yet that is essentially what we hear when the practicing physician proclaims to the world that he is a therapeutic nihilist.

Who would for a moment believe that a man devoted to the good of humanity, spending his whole life and energies in ameliorating their sufferings, sympathizing in their fortunes, and in every way within his power smoothing the rugged pathway of life, being at heart an anarchist or murderer of the deepest dye? Yet that would not be more inconsistent with human nature than for a physician to really be a therapeutic nihilist. I take the ground that therapeutic nihilism and the practice of medicine are equally as incompatible as is oil and water. Understand me, I do not deny that there are men practicing medicine today who are therapeutic nihilists, nor would I assume that there are not men preaching the gospel today who are at heart as black as hell, but the one is not a physician and the other is not a minister of the gospel in the true meaning of those terms. Yes, there are therapeutic atheists in the practice of medicine; in fact, a large number of them, but they are no more physicians in the true meaning of that term than is the Rocky Mountain tick an elk, simply because it happens to be sucking the blood of that royal animal, vampire like, and assuming the airs of the animal upon which it mercilessly feeds! Some one will say that is strong language. So it is, and the subject is one that demands language stronger than any in the English language with which I am acquainted.

But we will return to the essay mentioned, and from it make certain extracts and quotations, when we will again take up the thread of discussion:

"Dr. John H. Musser; * * * just as the present compares with twenty years ago, one can see less and less of the use of drugs. Treves; * * * the habit of taking medicines lies deep down in the hearts of the people. Not only do our patients crave active and militant protection and relief in the hour of suffering, but the physician knows through the teachings of practical experience that drugs are his legitimate and often trustworthy weapons of warfare, the strongest shield he has to interpose between his patients and the fell destroyer. Closely allied to the air, food and water apostles are the therapeutic nihilists, who, like Professor Osler, believe in the doctrine of self-limited diseases and look on while nature and the disease have it out. Dr. Jacobi calls this 'a sin of omission, which frequently rises to the dignity of a crime.' * * * Another most regrettable feature of present-day medicine is that public confidence in our professional efficiency is on the wane, as the frequent attacks in the lay press will well attest. * * * The normal results of this campaign are that a multitude of innocent people are increasingly driven into the hands of quacks; that systems of practice based on mysticism and misrepresentation are steadily gaining ground, and that patent medicine vendors are accumulating untold wealth at the expense of the unwary. * * * Drugs, with the exception of quinine in malaria and mercury in syphilis, are valueless as cures. —Billings. * * * Although the most important of all, and the one branch through which the public, the whole world, in fact, gauges our actual professional efficiency, therapeutics has ever remained a mere art. A remedy is still given because it has been found more or less efficacious in this or that condition by others, and the subdivisions headed 'treatment' or 'therpeutics' in our text-books are mere catalogues of drugs which are stated to be 'particularly useful,' 'most efficient,' 'very valuable,' 'commonly employed,' 'of great value,' etc., in this or that disease, and in which not an inkling is afforded as to *how* the remedy antagonizes the morbid process. * * * Are we justified in attributing to ignorance this loss of faith in a system of administering remedies which is irrational and archaic as the other departments of medicine are precise and modern? Indeed, it is merely because pharmaco-therapy has not kept pace with the immense strides of all other branches; that it is, with many physicians, 'almost moribund.' * * * Is there any indication that physiology will furnish the data we need to place pharmacology on the sound basis we so earnestly crave? I must frankly express the belief that, judging from present indications, the prospects are very discouraging. * * * They overlook the one source of information through which their own labors could be made promptly to bear fruit, that covered by the assertions of the great Russian physiologist, Professor Powlow, that in many instances 'the physician gives a more correct verdict concerning physiological processes than the physiologist himself,' and that 'clinical observation will consequently always remain a rich mine of physiological facts.' * * * Time has only served to sustain this statement, and to emphasize my own conviction that medicine, and particularly pharmacology, will not be redeemed from its present deplorable position as long as it will depend entirely upon physiologists for the elucidation of function. * * * A generation ago therapeutics was an art promising to develop into a science. At present it can not be classed as an art, nor as a science; it can only be classed as a confusion.—Professor Sollman. Need I further urge that new and more fruitful lines of thought are imperatively needed if the true worth of our profession, its humane purpose, and its very existence is to be perpetuated. * * * All true science begins with empiricism.—Huxley.

"Blest is he who knows no meaner strife
Than art's long struggle with the foes of life."—Holmes.

"It is for us to build. We may speak of confusion today; but confusion likewise prevails before each pillar, each stone, of any great edifice has found the place assigned to it by the co-ordinating mind of the architect. *Therapeutics needs architects and builders today far more than it does experimenters;* and when that fact will be thoroughly apprehended a new era for medicine, as a whole, will begin; one that will ultimately lead to the highest of human accomplishments—the mastery of diseases in all forms."

Such is the arraignment of our professional ability and our scientific learning of which so many boast by the leaders of the profession! Can any conscientious physician read over these quotations, of which there are thousands more in medical literature, and not feel the blush of shame mantle his cheek, and he be forced to cast down his eyes to the ground in humiliation; and is not the therapeutic nihilist self-convicted of fraud and deception, in that he appears to stand before men as a representative of a science and an art that in his own mind he thinks does not exist? *I take the ground, that if there exists such a thing as therapeutic nihilism in the mind of any physician, then it exists as a direct result of either wilful ignorance or selfish egotism!* I, an humble and isolated member of the medical profession, take that stand, and I challenge refutation from even the most learned and famous. I will say further that there exists today a science of therapeutics, and that science is within the reach of all. It is not found by a "favored and pampered son" who is the captain of the football team in the university. It is not revealed to the lens of the microscope, nor to the test tube of the chemist. It lies far and away beyond the ken of the experimental physiologist. The boasted laboratory diagnostician knows nothing of its beauties and simplicity. But, to the plodding empirical clinician, the bedside worker, the man who daily and hourly meets and conquers the "grim reaper" it is a rock of salvation to him and a star of hope to his suffering patient. He it is who is the wearer of a crown set with brilliant jewels snatched from the clutch of suffering and death. He plods along his weary way, little heeding the petty storms of egotism that lash into billows the sea of medical science, and he smiles in self-congratulation when he sees his dose of medicine accomplish the desired and expected results with as much precision as does the rifle ball from the gun of the expert marksman strike the center of the bull's-eye. The "tempest in a teapot," called medical politics, does not trouble him. He is not seduced nor besmirched by any of the numerous fads and foibles with which the more egotistical members of the profession are led astray. And all the medical knowledge which he possesses is within the reach of every practicing physician, and it is free to every one who will delve into the depths of the mine of wisdom and bring forth the diamonds therein deposited.

Knowledge is a jealous goddess, only bestowing her favors on the few who are willing to labor long and hard for the prizes which she bestows. She is not in any way influenced by political pulls nor college favoritism, nor is there a "royal road" to her smiles. I would not for worlds be understood as in any way decrying education per se, but education alone is not sufficient to give one absolute faith in the action of medicines, and certainty in their administration, that knowledge which we assemble under the term, therapeutics. There exists such a knowledge, and it may be attained by all. It may not be complete, neither is there any other science that is complete; but it is sufficiently complete to render the practice of medicine at large far more successful than it is at the present day. Let the mortality rate of many of the most prevalent diseases of the present day stand forth and give evidence to the world of the total inefficiency of the popular methods, and with them include the "laboratory diagnostician" and the "laboratory therapeutist." The mortality in pneumonia is greater today than it was thirty or fifty years ago, and so with many other diseases, in perhaps a lesser degree. Is it not time that physicians should stop and consider why this condition exists? The people generally are aware of this deplorable condition of affairs medical, and as a result they are rapidly drifting into the hands of the Osteopath, the Eddyite and the numerous other cults with which our country is rapidly becoming inundated. If the medical profession fails to stop and intelligently consider these matters, and as a result of that consideration face about and show to the people that the *cure of disease* is far more important than is the post-mortem verification of a scientific (?) diagnosis, then the people will entirely withdraw their patronage, and the members of the medical profession will be forced to find pastures new.

But, to attain a knowledge of the science of therapeutics. Let the man who would attain to that degree of perfection in his profession stop in his tracks, as it were, and begin again to ascend the hill of medical knowledge, and, as he climbs, let him remember well the foundation principles upon which is erected the whole superstructure of medical science. These truths were perhaps taught to him as a student, or at least should have been, but when he had obtained the coveted diploma and launched out as an independent man he was led to forget them because he was not aware that they must govern his practice by their eternal principles. Another point to be acquired by the would-be therapeutist: He must possess the power to reason along exact logical lines, from cause to effect, and from effect back to cause again. Then he must be a close observer of nature in all her manifestations, especially so when those manifestations are to be read on the human body. Now, with a mind trained in this way, and with a mind "as a little child," let the good work begin, and the harvest will be, indeed, rich.

*Disease is wrong life,* is the first principle upong which the other foundation stones must rest. Conversely: Any remedy employed for the cure or amelioration of disease must be one whose action looks toward the righting of that wrong, and must never in any way impede the existing efforts of nature to right the wrong, but they must act as helpers or assistants to the efforts of nature. The highest pinnacle of professional attainment is that of intelligently assisting nature, never impeding her efforts in any way. He who presumes to direct instead of assisting will always become an assistant to the grim reaper; so he has the privilege of choosing which he will assist. The efforts of nature to right the existing wrong, and perhaps in her endeavor to accomplish that end she shows certain evidences of that effort, to which we have given the term disease, and the accomplished therapeutist must intelligently interpret those conditions, and in doing so he must be competent to reason intelligently. Should one be leisurely walking along a quiet roadway, and suddenly a peculiar sound is brought to his ear, if he can reason connectedly, he will at once follow about this line of reasoning: On the waves of the atmosphere there has been brought to the eardrum the result of the sudden oxidation of a chemical mixture commonly called gunpowder; that ignition taking place in a restricted or confined area, commonly called a gun; therefore, the sound that I have heard was a gunshot. I have heard it many times before; therefore, I need feel no alarm as to its cause, nor need I fear the results. Now, how many men sit at the bedside of the suffering patient and reason out the existing conditions, and the remedy to employ, in such a connected manner as this? I fear that no man who is an avowed therapeutic nihilist ever once in his life performed such a feat of mental gymnastics. If he had ever done so, then he could not be a medical abortion, provided he had the other requisite mental attainments. To properly interpret these signs of existing disease, one must possess a good working knowledge of the science of pathology and morbid anatomy. To acquire that, let him take up the study of the standard works on that subject, together with the facts of physiology. I am one of "the old-timers," and, in my day, Virchow served as a basis, and with that at that time the standard works, Green's Pathological Anatomy and Williams' Principles of Medicine, associated with Carpenter's Physiology, was sufficient foundation upon which to begin the erection of the superstructure of therapeutics. I do not mention these particular works with the idea of advising the present-day student to procure and study these same books, but I mention them as illustrative of the direction in which one must travel to reach the goal. Let the student of the present day study the books of more modern date, but always with the assurance that those old books were sufficient in their day to enable many men to successfully cope with disease; yes, equally as successfully as do the more modern books.

Now, the explosion of the gunpowder in the instance mentioned always produces the same kind of sound. But the character of the sound depends somewhat on the kind of gun in use; thus, many can readily tell from the sound whether the firearm used was a pistol, a rifle, a shotgun, etc. So it is in diseases. Like causes always produce like effects, but those effects are somewhat modified by the existing conditions. This explains why we never see two persons each of whom are suffering from a given cause of disease, manifesting the same identical picture of disease. The conditions in the several persons are different; therefore, the different phenomena. Yet the cause is identical. Thinking along this line shows us plainly that we can not successfully apply the same treatment in the different cases, notwithstanding the fact that the cause of the disease was the same in the different cases. Thus we see that every man must do his own thinking, if he would successfully cope with disease. He can not take the word of a man living perhaps in the antipodes, but the brain power at the bedside of the patient must be employed to bring about a cure. "Favorite prescriptions" are a delusion and a snare to a physician, and a pitfall to the sufferer, and the bottom of the pit is ofttimes "six feet due east and west, and six feet perpendicular." Volumes have been and might be written on this division of our subject, but perhaps sufficient has been said to point the way, dimly, to the would-be inquirer and learner; so we must pass to another division of the subject.

*Every medicine or remedy employed for the cure or amelioration of disease is a dynamic force, invariably becoming a cause that produces a given effect, conditions being equal.* Thus we have disease as an effect of a given cause; now the therapeutist must apply a cause that will neutralize the given disease, or, the effect of the remedy must be such as will produce the condition known as health. *Every therapeutist must know the action of the medicine he employs.* If he has been long in the practice of medicine, and has not practiced blindly, he will have acquired a vast fund of information relative to the action of medicine, as a result of his numerous observations; but, if he is a new "Richmond in the field," then he will be compelled to take his information second hand. Again must I illustrate by citing the methods used in the beginning, in the days when I was a tyro. Again will I cite old books, and I really do not admit senility, but I sometimes question if the more modern books are essentially a material improvement over those old standard books. They at least produced a very considerable per cent of successful physicians, and what more can be said of those more modern? In the old days, we would likely begin the study of the action of medicines in "Headland on the Action of Medicines." What a rich and vast field of information it contained. Then, we might take up the study of remedies proper in Wood's Therapeutics, that classical work that is now in, I believe, the fourteenth edition. To the man who desires to honestly master the action of medicines, so far as it is possible for the finite mind to master that subject, will find in these two books, or similar ones, all that his mental capacity cares to assimilate, unless he is so fortunate as to be possessed of a mentality beyond that of the average mortal. Let him take up any one of the more common medicines. Veratrum, we will say, as an example. Headland will tell him all about how it enters the system, and how it is carried to the part upon which it acts; then Wood will tell him the tissue upon which it acts, and as much as is known about how it acts. In the quotations at the beginning of this essay, one of the excuses offered for the existence of therapeutic nihilism was that experimental physiologist could not tell *how* a given remedy did its work. Would it be rational for a man to walk off a high precipice simply because he was unable to tell *how* the law of gravitation would cause him to be dashed to pieces on the rocks below? One hypothesis is as reasonable as the other; the true therapeutist cares but little for the details of the corpuscular changes produced in a given atom; it is the final result that cures his case. It is true, such knowledge constitutes a beautiful accomplishment, but it is decidedly more ornamental than useful. Now a vision comes to me. I see the readers of this humble essay holding up their hands in holy horror, crying out, stone him; he advocates pure empiricism!

Yes, I do, and will hurl back into the teeth of these "holier than thou" would-be scientists this inquiry: Have you anything better to offer, and, if you have nothing better to offer, would it not be more graceful on your part if you intelligently used this empirical knowledge, and would it not be better for your unfortunate patients and for the reputation of the medical profession at large and yourself in particular, if you would use this knowledge, instead of hiding your ignorance behind the cloak of medical or therapeutic nihilism? Huxley tells us that empiricism is the beginning of science. You as a medical nihilist have not even that beginning of which to boast. The average "science" is simply a mass of fads and fancies, seized upon and fostered because of the seeming "authority" from which they appear to arise, while very often that "authority" is simply a commercial boost with no absolute reason for its existence save the avarice of its promulgator. In the instance of veratrum, before mentioned, it serves as an illustration that is applicable to every drug in the materia medica, as well as to every method employed to cure disease by an intelligent physician. Even the cures produced by the ridiculed cults, and there are some cures, are brought about by means and methods that can be analyzed out and explained on such inductive and deductive reasoning as in the instance of the sound of the gunshot and the action of veratrum. But such knowledge can only be obtained at the expense of mental effort, persistence and discrimination. It is a shame that so many men are willing to tamper with human life upon the basis of information ladled out to them by the ubiquitous "detail man" of some enterprising manufacturer. It is high time that the mentally industrious of the medical profession united themselves into solid body, and drew distinctly the line of demarkation separating them from the mass of parasites who are too lazy to study and too obtuse to learn if they did study. Their "think shop" is decidedly out of repair, or perhaps was never in good working order. In the application of this line of study, it must be distinctly understood that medicines of established and unquestionable purity must be employed, else failure will be sure to follow. None of the ordinary "slop" appearing under the ordinary labels will bring success. The medicines must come from a reputable manufacturer, and their activity must be beyond dispute.

As before stated, every medicine when introduced into the human body is a dynamic force, and that force is governed by law as inflexible as the law of gravitation. If any one doubts that statement, let him try the action of any reliable preparation of any well known drug, anywhere along the line from castor oil to aconitine, on himself, if not other subject is available, and from the moment he gives the drug a fair trial he will be compelled to admit the truth of this statement. Any intelligent person can learn these laws, but he will not learn them in the physiological laboratory; he will learn them at the bedside. The human body is not a test tube nor an incubator in so far as the action of medicines is concerned. A medicine acts far differently when applied to a diseased structure, as compared to its action on the same structure when in a normal condition. No remedy given to an anesthetized dog will indicate with any degree of certainty in what line of troubles it will act on the diseased human being and bring about a cure. Disease must not be viewed as a distinct entity. Its cause may assume that form, but disease is a wrong of human tissues, either functional or structural. The cause may be attacked as an intruder, but the disease itself is a wrong method of life, involving the whole or a part of the body. Perhaps the body is endowed with auto-protective powers, and if it is the duty of the physician is to encourage its efforts of self-protection, never impeding in any way those efforts, but intelligently seconding them.

All disease processes are governed by laws as inflexible as the "laws of the Medes and Persians." No variation is ever found. Let the therapeutic nihilist learn those laws, which he can do by an unbiased study of pathologic processes, then apply his remedies according to the laws by which they are governed, and who will say that the results will not be all that could be wished for by even the most exacting?

Call this empiricism if you will, but until so-called science can give us something better, I for one am very well satisfied to follow the empirical lead. I know a man who has been in constant and active practice for a period of twenty-seven years, doing sufficient to have accumulated a snug fortune, and in all that time he has written death certificates to the surprising figure of eighty-six. Let the therapeutic nihilist stand up and tell us what his record has been for the same length of time, doing the same amount of work. If empiricism gives us such results as this, then let the ultrascientific therapeutist or nihilist forever after hold his peace. These men who so loudly proclaim their medical atheism are a stench in the nostrils of the honest, conscientious physician, and are a parasite on the professional body, sucking its blood, disfiguring its beauty, hampering its usefulness, and are self-confessed frauds and swindlers.

Now let us reconsider the title of this essay, and also reflect on the quotations cited in its first portion. The information hinted at in this article is obtainable by any one who will diligently seek it. Science offers nothing better. If these people who are therapeutic nihilists have not this information or knowledge, crude and limited though it be, then their ignorance must be ascribed to a lack of desire on their part. We must call that simple, old-fashioned laziness. If they think that they do not need this information, deeming themselves sufficiently wise without it, then they are egotists, and a man in the medical profession who is an egotist is a dangerous person to be at large. God speed the day when it will be considered equally as disgraceful to be classed as a medical or therapeutic nihilist as it is at the present time to be known as a swindler or a thief.

Volumes might be written along the lines of each division of thought as hinted at in this essay, but, if what has been written should induce one single poor doubter to hesitate, turn about and follow the glimmering light of humane and successful therapeutics, the object of its author will be most liberally accomplished.

